# Pain-Track: a time-series approach for the description and analysis of the burden of pain

**DOI:** 10.1186/s13104-021-05636-2

**Published:** 2021-06-05

**Authors:** Wladimir J. Alonso, Cynthia Schuck-Paim

**Affiliations:** 1 Epidemiology Research Group EPIDOT, Department of Public Health, Federal University of Santa Catarina, Florianopolis, Santa Catarina Brazil; 2 Welfare Footprint Project, Murcia, Spain

**Keywords:** Pain, Time-series, Pain scales, pain profile, quantitative measurement, Burden assessment

## Abstract

**Objective:**

To present the Pain-Track, a novel framework for the description and analysis of the pain experience based on its temporal evolution, around which intensity and other attributes of pain (texture, anatomy), interventions and clinical symptoms can be registered. This time-series approach can provide valuable insight on the expected evolution of the pain typically associated with different medical conditions and on time-varying (risk) factors associated with the temporal dynamics of pain.

**Results:**

We illustrate the use of the framework to explore hypotheses on the temporal profile of the pain associated with an acute injury (bone fracture), and the magnitude of the pain burden it represents. We also show that, by focusing on the critical dimensions of the pain experience (intensity and time), the approach can help map different conditions to a common scale directly relating to the experiences of those who endure them (time in pain), providing the basis for the quantification of the burden of pain inflicted upon individuals or populations. An electronic version for data entry and interpretation is also presented.

**Supplementary Information:**

The online version contains supplementary material available at 10.1186/s13104-021-05636-2.

## Introduction

Given the many challenges in the direct assessment of the pain experience [1–3], several scales were developed to evaluate its intensity as perceived by patients, often by means of self-reporting questionnaires [4, 5]. These instruments find widespread use in clinical and research settings [4]. However, while greatly useful to represent the perceived intensity of pain, these scales are not designed to capture two important elements of the pain experience: its duration and pattern of evolution. Like many other biological phenomena, pain is a dynamic process that unfolds along a temporal dimension: it may develop and resolve gradually or suddenly, be brief or prolonged, constant or episodic. Yet such a critical component has been often assigned an accessory role in the assessment of pain. Temporal profiles of the pain typically associated with different injuries and diseases have been seldom studied [6]. Consequently, the possibility to examine temporal relationships between pain and other time-varying variables (e.g., risk factors), patterns in the development of pain and the effectiveness of therapeutic protocols over time has also been constrained.

We present an operational framework for the description and assessment of pain as a time series, which we refer to as Pain-Track. As the name implies, it tracks the evolution of pain intensity over time, based on its temporal unfolding along a continuous axis, around which intensity and other attributes of pain (texture, anatomy, clinical symptoms, interventions) can be chronologically placed. Like with other dynamic phenomena, the use of a time series approach can provide valuable insight and modelling potential. In the case of a sensorial experience like pain, it can also foster the explicit representation of hypotheses for the evolution of the pain typically associated with specific medical conditions, and the investigation of the extent to which pain patterns match underlying pathophysiological processes. The approach also enables quantifying the total time spent at different levels of pain intensity due to one or more conditions, thus offering a quantitative measure of the burden of pain experienced by individuals (and, as we will argue, populations), based on a universal and meaningful metric (time). We illustrate the framework to explore hypotheses on the temporal profile of the pain associated with a bone fracture, and quantify the magnitude of the burden it represents. An electronic version for data entry is also made available. Before introducing the framework, we describe the rationale behind the use of four reference levels of pain intensity.

## Main text

### Reference levels of pain intensity

Continuous scales of pain intensity have been widely used [5, 12]. However, given the abstract nature of numerical scores, numerical ratings are often inconsistent across individuals [4], and force ratings into a linear scale of pain intensity even though the sensorial equivalence of pain between successive unit divisions is unknown. Conversely, in verbal rating scales patients choose descriptors of pain intensity close to those used in colloquial speech. Discrete categories (e.g., ‘mild, moderate and severe’ [2]) provide more relatable grading systems, arguably keeping subjective variation within narrower limits (as the terms -albeit ambiguously- have a self-contained meaning, without requiring the abstraction of ranges and scales).

Still, categories should be defined as precisely as possible (e.g.,‘moderate’ is an elastic term, with likely different meanings to different patients), anchored on specific criteria to reduce ambiguity and increase consistency of reporting. Here we guide the definition of intensity categories by specific criteria, grounded on (evolutionary) principles that should be common to most pain experiences: the disruptive character of the pain experience and its effectiveness to promote adaptive behaviors. Pain is an adaptive warning message of actual or potential danger that must be loud enough to change behavior and reduce the likelihood that survival and reproduction are compromised [3, 7]. The greater the threat, the louder this signal should be to ensure it will take precedence over other bids for behavioral execution [8]. Accordingly, more unpleasant sensations should be in general more disruptive [9]. For example, the degree of unpleasantness associated with severe lack of food, impaired oxygen intake, and imminent dangers should be high enough to ensure that less critical ongoing behaviors are put on-hold until the threat is reduced. The same applies to endogenous threats, as changes in behavior (e.g.resting) are an important part of a strategy to enable healing [10]. A positive association between pain intensity and the degree of disruption (the extent to which attention to other tasks and ongoing behaviors are affected) is thus expected. This association is also expected from a mechanistic perspective, as higher pain intensities are likely to interfere with the attentional processing of other tasks [11, 12] and cues [13]. We use these criteria to reduce ambiguity in the classification of pain intensity and establish thresholds among four reference categories (described in Table 1). We intentionally avoid the terminology mild, moderate and severe, as it has been used in multiple contexts with different meanings, using instead terms that evoke an empathic appreciation of intensity. Since the four levels are divisions imposed on a continuum, there is no theoretical limit to further increases in resolution.

**Table 1:** Reference categories of pain intensity

Category	Definition
Excruciating	Threshold of pain under which many people would choose to take their life rather than standing the pain. This is the case, for example, of severe burning events, which may make victims jump from buildings, or other conditions associated with suicidal attempts by sufferers (e.g., cluster headaches). Many forms of torture have been designed to inflict pain at this level. Behavioral patterns can include loud screaming, involuntary shaking and extreme restlessness
Disabling	Most forms of functioning or enjoyment are prevented as the direct result of pain. Symptoms are continuously distressing. Individuals affected often substantially reduce activity levels and refrain from moving. Pain at this level can disrupt or prevent sleeping. Only strong analgesia can relieve it
Hurtful	Pain experiences that most would consider disruptive of daily routine. Although not entirely preventing individuals from functioning, their ability to do so is impaired as the direct result of pain, and often accompanied by the desire to take painkillers or seek treatment. Frequent complaints are often present. The possibility to enjoy pleasant experiences is impaired, as is performance on mentally demanding tasks, alertness and attention to ongoing stimuli
Annoying	Pain experiences are not intense enough to disrupt the routine or daily activities of individuals, their possibility to enjoy pleasant (positive) experiences, or their ability to conduct mentally demanding tasks that require attention. Sufferers do not think about this sensation most of the time, and when they do they can adapt to it

### Pain as a time series

**Fig. 1:**
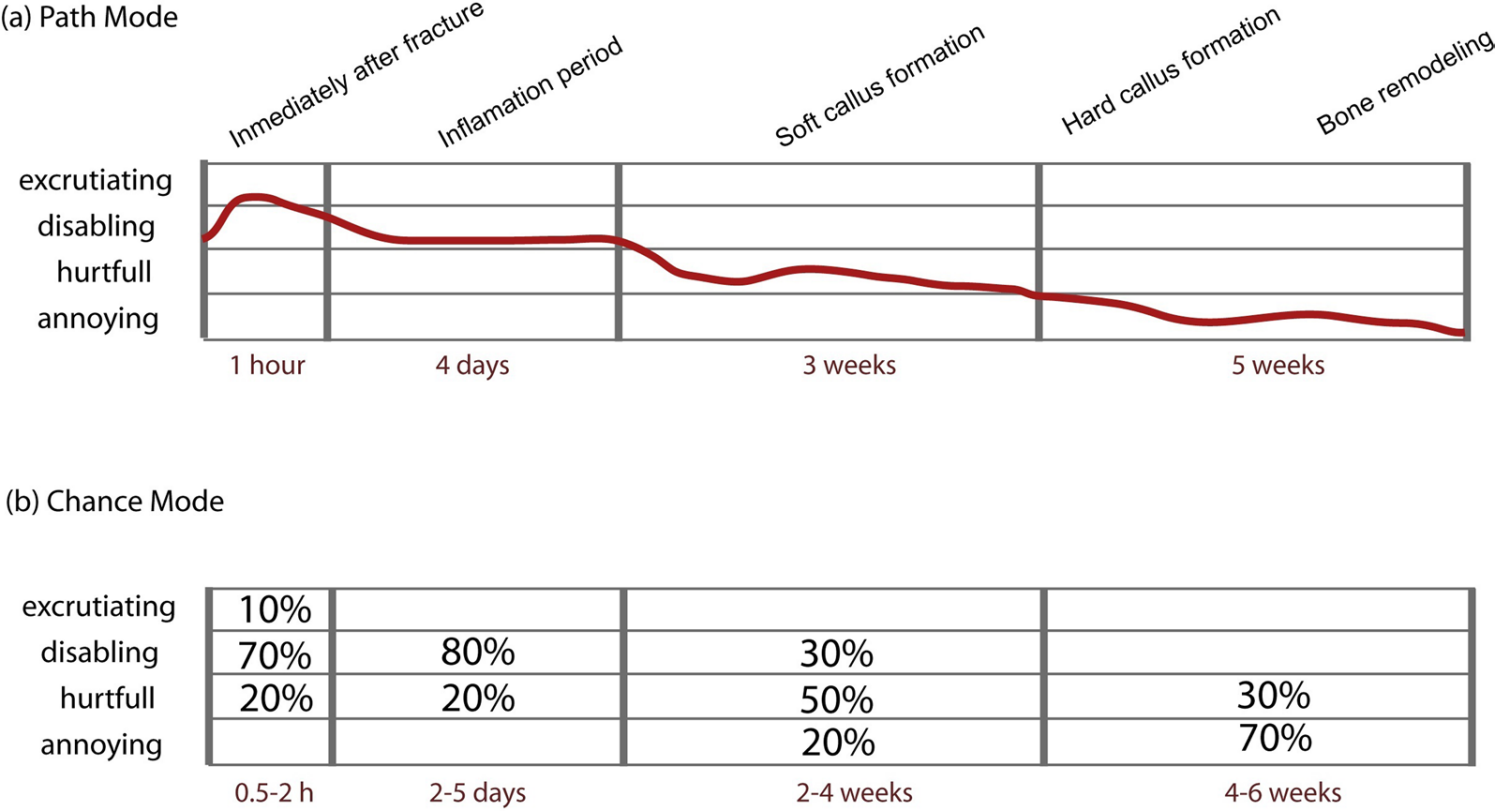
Clinical application of the Pain-Track framework to describe the temporal evolution of the pain associated with a hypothetical leg fracture. No therapeutic intervention is assumed. Pain intensity is represented in the vertical axis and the range of probable durations is shown below each time segment. Since pain experiences can unfold over a wide range of periods, time segments represent different durations to ensure the flexibility needed. **a** Path mode: hypothesized temporal evolution of pain for a patient; **b** Chance mode: time is partitioned into discrete segments used to delimitate intervals when pain intensity changes. Percentages can represent either (i) the percentage of the population that feels pain at the level or (ii) the probability that the pain belongs to that category of intensity. Here, percentages should be interpreted as in (i) (expected temporal profile of the pain associated with leg fractures at the population level). For simplicity, the possibility that chronic pain develops is not depicted. Intensity and duration values are justified in the Additional File 1

The binding hub for data capture, visualization and analysis is a standardized visual framework where pain is represented as a time series. In Fig. 1 we use it to describe the expected temporal evolution of the pain associated with a hypothetical leg fracture.

Two are the possible ways to register the evolution of pain intensity: the path (Fig. 1a) and chance (Fig. 1b) modes. In the path mode, pain intensity levels are represented by a continuous (though not necessarily linear) gradient from no pain to excruciating pain. Estimates of variability (eg, confidence intervals) can also be added. This mode is a convenient way of recording pain in the clinical setting. The chance mode (Fig. 1b) is designed to capture (i) the expected temporal profile of pain at the population level, considering the expected variability in pain perception in a population or (ii) situations where uncertainty in the classification of pain intensity is present, a possibility useful for assessing pain in non-verbal subjects. Accordingly, for each time segment, intensity categories in each column can be filled either (i) with the estimated proportion of the population that experiences pain at each level or (ii) with the probability that the pain belongs to each category of intensity. If stacked cells do not add up to 100%, the remainder percentage is attributed to a state of ‘no pain’. To incorporate uncertainty in the duration of time segments, each is represented by a confidence interval.

Justification of the estimates in Fig. 1 is provided in the Additional File 1. Briefly, the initial period represents the sharp, piercing pain often described by patients at the time of fracture [14], when mechanosensitive nerve receptors are activated. At this time, pain is most likely of a disabling nature (Fig. 1a), capturing nearly all the individual’s attention: sufferers are unable to perform other activities and strong analgesia is commonly required. The chance mode (Fig. 1b) captures the possibility that a small percentage of patients (10%), with a low pain threshold, experience excruciating pain, based on reports that some patients beg to be sedated or have their limbs amputated [15]. Once the fracture is aligned or stabilized, the sharpest pain is commonly replaced by a dull, sustained pain that would last some days in the absence of analgesic treatment, coinciding with the peak of the inflammatory process [16]. Pain typically subsides during soft callus formation. This period usually lasts 2–4 weeks, from stabilization of the inflammatory process until formation of the hard callus and initiation of bone remodeling. At this stage, the expression of osteoinduction mediators at the injury site, particularly members of the bone morphogenetic protein (BMP) family, underlie the persistent pain that some patients report (BMP2 has been linked to pain pathways [17], inflammation [18] and release of neuroinflammatory proteins [19]).

The structure of the Pain-Track offers a means to explore putative associations between pain patterns and temporal variation in brain activity (e.g. [6]) or other continuous parameters that may become available [3]. It accommodates data collection processes (retrospective or real-time) conducted with traditional instruments and time-indexed information, over which pain experiences can be anchored. The proposed notation has been designed to be of easy use by clinicians, patients and researchers, and amenable to digital capture and processing. The simplicity of this method also allows for patients to self-record pain episodes, which can improve accuracy compared with later recall [20]. To facilitate the use of the framework, an electronic version was also developed, freely available at http://pain-track.org.

The use of the Pain-track framework seems similarly promising for the description of conditions leading to physiological discomfort (e.g. hunger, thirst) or psychological pain (e.g., anxiety, depression). The degree to which behavior and attention to other ongoing experiences are disrupted by these experiences can be used as a yard-stick to infer the intensity of the sensation.

### Quantifying the burden of pain

By focusing on both critical dimensions of the pain experience (intensity and time), Pain-Tracks can map different conditions to a common scale: time in pain. This enables quantifying the cumulative load of the painful events experienced [21, 22], namely the sum of the time spent in pain at each intensity category as a result of the conditions examined.

**Fig. 2:**
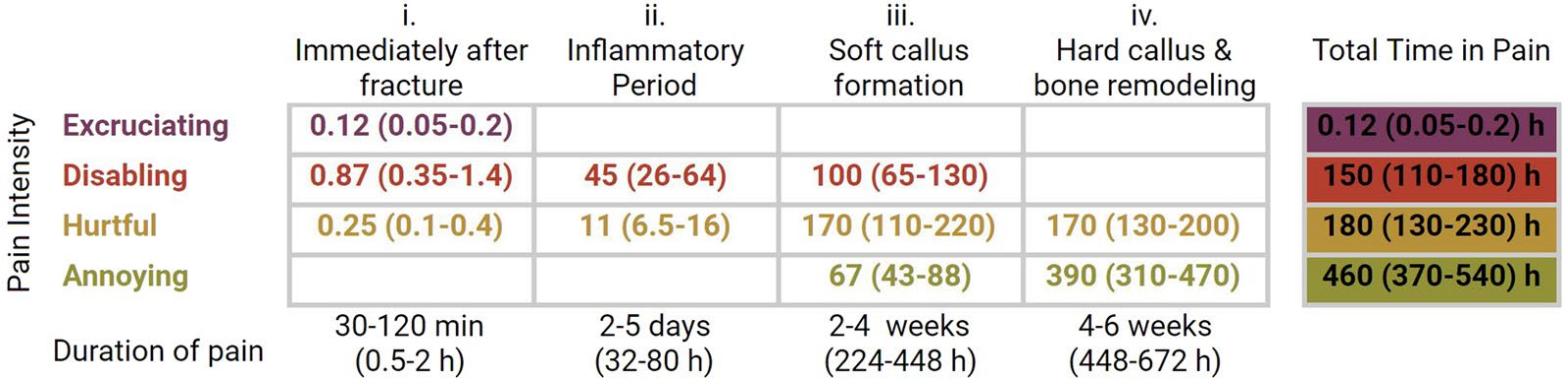
Expected average time (hours) in pain (95% confidence interval) at each level of pain intensity due to a type of leg fracture. Parameter values (pain intensity and duration) are depicted in Fig. 1B. The uncertainties associated with the duration of each segment were propagated with a Monte Carlo simulation [34], assuming a gaussian distribution for the duration interval. The expected times in pain endured by the average member of a population due to the fractures can be determined by weighing the total times by the estimated fracture prevalence. For illustrative purposes, assuming a prevalence of 5–10%, expected times in excruciating, disabling, hurtful and annoying pain by the average population member are, respectively: 0.2 (0.2–1) minutes; 11 (6.5–16) hours, 13 (7.6–20) hours and 33 (22–48) hours

For example, from the parameters in Fig. 1b, it is possible to estimate the time in pain at each intensity level that individuals sustaining this leg fracture are expected to endure. If, hypothetically, the first segment lasted precisely 60 min, then 6, 42 and 12 min of pain at the excruciating, disabling, and hurtful levels would be expected, respectively, during this period (e.g. 60 min × 10% of the population experiencing excruciating pain = 6 min). The same procedure can be conducted with all segments, and results added up to determine the total time in pain at each category (Fig. 2).

At the population level, the expected times in pain endured by the average member of a population can be determined as the product between the resulting times and the estimated prevalence of the condition in that population. Although pain is a concept that inherently concerns individuals, analyses at the population level enable comparing the burden of pain imposed by different injuries and diseases, and how it varies across demographics, geographies and time.

It is easy to appreciate that reducing the time individuals spend in pain of any intensity will improve well-being. However, to what extent can comparisons of time in pain be made among different categories of pain intensity? One way to determine the overall burden of pain would be by aggregating pain intensities of different categories into a single metric. Approaches rooted in this concept have been indeed used to determine the burden of diseases, combining the time spent in the disease state with its severity (disability) [23]. In the present case, however, such an exercise requires understanding the numerical relationship among the intensity categories in terms of the aversiveness they cause: how much worse is the hurtful experience compared to an annoying or disabling pain?; or how long should an individual endure an annoying pain to make it equivalent to a few minutes of excruciating pain? Given the current lack of knowledge and means to address these questions, the analysis of the total time spent in each category of pain intensity represents a more accurate and transparent approach, grounded on explicit parameters with clinical meaning. Importantly, by keeping intensity categories disaggregated, no information is lost. This approach might prove to be more informative, as it enables the assessment of the impact of different conditions along a scale of negative experiences.

## Limitations

As with other scales of pain, Pain-Track registries must be inspected with the awareness that noise, biases and confounding factors will blur access to a realistic and accurate depiction of the pain experience. Additionally, future research is needed to test the psychometric properties (validity and reliability) of the Pain-Track in the clinical setting.

## Supplementary Information

**Additional file 1**. Clinical applications of the Pain-Track framework: temporal profile of pain.

## Data Availability

Not applicable.
