# Supplementary material for: Pain-Track: a time-series approach for the description and analysis of the burden of pain
Source: BMC Res Notes. 2021 Jun 5;14:229. doi: 10.1186/s13104-021-05636-2 (PMC8180079; doi:10.1186/s13104-021-05636-2)
Supplement: Supplementary file 1 — Additional file 1. Clinical applications of the Pain-Track framework: temporal profile of pain. [file 13104_2021_5636_MOESM1_ESM.pdf]

Additional File 1. Clinical applications of the Pain-Track framework: temporal profile of pain

Pain-Track: a time-series approach for the description and analysis of the burden of pain (Alonso & Schuck-Paim)

Clinical application of the Pain-Track framework for the description of the temporal evolution of the pain associated with a typical leg fracture (Figure S1). The Pain-Track was filled, and the parameter values justified, by Dr. Pedro E Salinas Salazar, MD, pediatrician and anesthesiologist, executive chief of the Anesthesiology Service of the Hospital Universitario Dr. Angel Larralde (associated with the Instituto Venezolano de Los Seguros Sociales, IVSS). The top panels of Figure S1 (path mode) illustrate an expected practical use of the Pain-Track in the clinical setting, to record the temporal evolution of the pain, as inferred by the clinician or reported by the patient. The chance mode (bottom panels) depict the hypothesized profile of the pain associated with these conditions at the population level (percentages correspond to the expected proportion of the population that endure pain at each level).

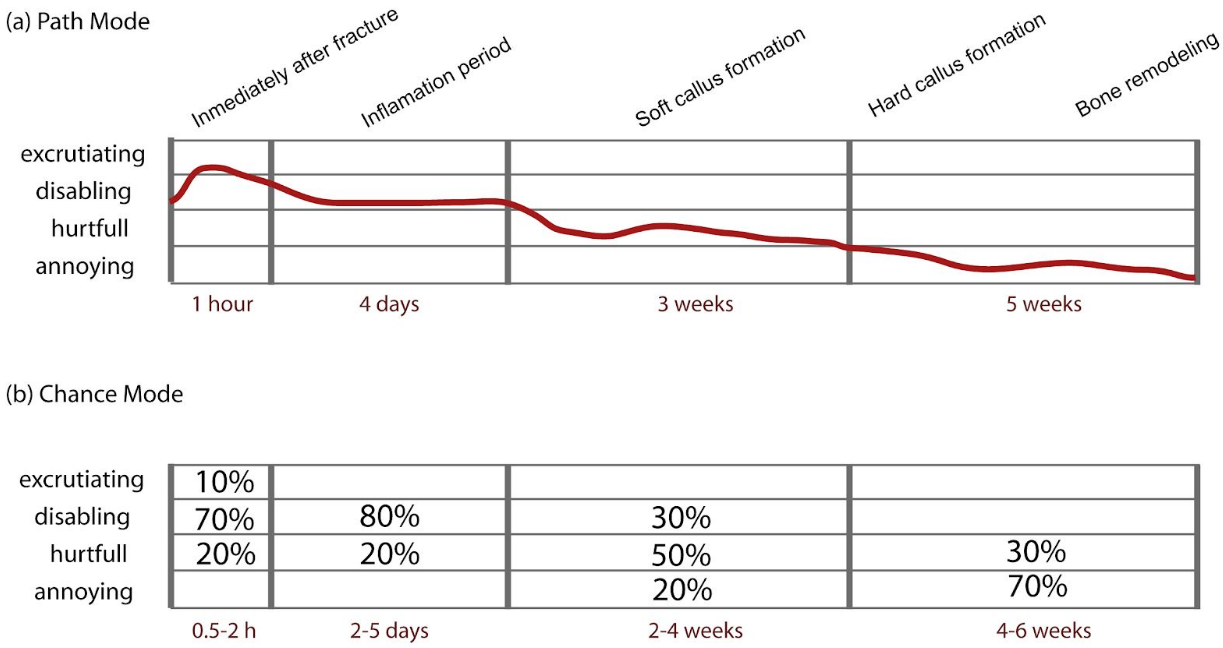

Figure S1. Clinical application of the Pain-Track framework to describe the temporal evolution of the pain associated with a leg fracture. For illustrative purposes, in the path mode no uncertainty or variability is attributed to the duration of the intervals. Criteria for the classification of intensity categories are described in the main manuscript text.

Figure S1. Justification of parameter values:

Segment 1 (pain immediately after the fracture): Immediately after the fracture, mechanosensitive nerve receptors are activated, sending nociceptive signals to the spinal cord and brain, which translate into the sharp, piercing pain that patients describe at the time of injury [1]. The periosteum has an extremely dense sensory and sympathetic innervation, where most of the mechanosensory fibers activated immediately after fracture are believed to be located [2]. The initial pain following a fracture is thus most likely of a Disabling nature: sufferers are unable to perform other activities and strong analgesia is required. A small percentage of patients (10%), with a low pain threshold, may even beg to be sedated and, in extreme cases, to have their limbs amputated [3]. The bone marrow has similar innervation to that of the periosteum, so its participation in the genesis of pain should also be considered. This pain-generating process can be reactivated by movements or mechanical distortions of the broken bone, which supports the mechanosensitivity role of the nerve fibers innervating the bone [4]. This period can last 30 minutes to two hours.

Segment 2 (inflammation period). After the fracture is aligned or stabilized, the sharpest, most intense pain is replaced by a dull, sustained, and aching pain. At this stage, the pain remains disabling, because even when the fracture is stabilized, the inflammatory process is at its peak and functional disability has not changed. This pain, unlike the one initially described, is mediated by the A-delta and C fibers that are also found in the periosteum, bone marrow, and mineralized bone [5]. Some authors consider that the demyelinated C-fibers are responsible for this pain, which would explain why their activation is not immediate [6]. This period is also characterized by the release of several proinflammatory substances, many of which work simultaneously as nociceptive transmitters [7]. The inflammatory period begins immediately after the injury, but the initial pain is activated by different mechanisms. During this period a hematoma appears around the fracture, which exerts a mechanical effect on the nerve fibers, consolidating the pain. However, within the hematoma, different cells start the healing process [8].

Segment 3 (soft callus formation): although this period is not the most painful (hence the gradual decrease in the probability of more intense pain categories), it is of enormous importance when it comes to the possibility of developing chronic pain in the future. When the fracture does not severely damage the nerves and heals properly, pain decreases with soft callus formation and the bone bridge disappears entirely with bone repair [9]. Soft callus formation is carried out by chondrocytes and fibroblasts, which are stimulated by the coordinated expression of several growth factors, such as transforming growth factor-β (TGF-β1 and β2), platelet-derived growth factor (PDGF), fibroblasts growth factor-1 (FGF-1), and insulin-like growth factor (IGF), along with other substances that promote cell proliferation and chondrogenesis [10]. These elements also participate in the activation of the painful path associated with the fracture [4]. These data

suggest that the molecular mechanisms involved in the early stages of bone repair are also involved in pain signaling. Although we now understand much more about the molecules released during the inflammatory response, their role in pain regulation is still unclear. Unfortunately, in some cases, fracture healing is inadequate and chronic pain develops. It is possible that ectopic and exuberant sprouting of sympathetic and sensory nerve fibers and the formation of neuroma-like structures are involved in the establishment of chronic pain in bones with impaired healing [11]. There is also the possibility that even when the bone heals properly, the mechanisms responsible for peripheral and central sensitization perpetuate over time, leading to the appearance of Complex Regional Pain Syndrome, or CRPS [12]. Through this mechanism, allodynia (normally innocuous stimuli perceived as painful) and hyperalgesia (noxious stimuli perceived as prolonged and exaggerated pain) can develop in patients with fractures [13]. At this stage the establishment of adequate analgesic treatment is crucial. The emergence of chronic pain, whether expressed as CRPS or hyperalgesia/allodynia, requires the convergence of chemical, electrophysiological, and pharmacological factors, so the ability to control at least one of these factors may prevent these complications. These concepts underlie the distribution of proportions attributed to each pain intensity level. Even though healing is already underway, and sufferers are able to function (hence the higher likelihood of pain at the Hurtful level), some patients are still unable to conduct their routinary activities or enjoy any form of entertainment (a definition compatible with the criteria in the Disabling level), which may be related to the formation of the soft callus itself or the possibility of a neurological disorder caused by the fracture. This period starts with the stabilization of the inflammatory process until the formation of the hard callus and the initiation of the bone remodeling process.

Segment 4 (hard callus formation and bone remodeling): in this period, consisting of two stages, the soft callus is replaced by a harder bone tissue which will be remodeled to its original configuration prior to the fracture. Two groups of cells act consecutively in these stages: osteoblasts (initially) and osteoclasts (at the end of the process) [14]. To activate the systems that lead to new bone formation and remodeling, some biochemical mediators are required, with the members of the Bone Morphogenetic Protein (BMP) family representing the most important. BMPs are a group of cytokines related to the transforming growth factors beta (TGF-β), of which BMP2 has shown the ability to activate some pain pathways in animal models of bone cancer [15]. BMP2 has been used clinically to perform arthrodesis and other traumatology procedures due to its powerful osteoinductive properties, but its use has been linked to local pain and inflammation associated with transient increases in thermal and mechanical sensitivity [16]. Several other studies have shown that BMP2 is able to stimulate other neuroinflammatory proteins which generate pain through different routes and can cause radiculitis, swelling of the cervical spine, and even seromas [17]. Therefore, the expression of BMPs at the injury site, as osteoinduction mediators, can explain the persistent pain that patients often report, even when the fracture is already stable. In the final stages of bone remodeling, patients almost have no pain, reporting frequently only a mild discomfort (hence the higher likelihood of Annoying level).

References

1. Mitchell SAT, Majuta LA, Mantyh PW. New Insights in Understanding and Treating Bone Fracture Pain. Curr Osteoporos Rep. 2018;16:325–32.

2. Mantyh PW. Mechanisms that drive bone pain across the lifespan. Br J Clin Pharmacol. 2019;85:1103–13.

3. del Rosario MA, Martín AS, Ortega JJ, Feria M. Temporary sedation with midazolam for control of severe incident pain. J Pain Symptom Manage. 2001;21:439–42.

4. Alves CJ, Neto E, Sousa DM, Leitão L, Vasconcelos DM, Ribeiro-Silva M, et al. Fracture pain—Traveling unknown pathways. Bone. 2016;85:107–14.

5. Mantyh PW. The neurobiology of skeletal pain. Eur J Neurosci. 2014;39:508–19.

6. Martin CD, Jimenez-Andrade JM, Ghilardi JR, Mantyh PW. Organization of a unique net-like meshwork of CGRP+ sensory fibers in the mouse periosteum: implications for the generation and maintenance of bone fracture pain. Neurosci Lett. 2007;427:148–52.

7. Woolf CJ, Ma Q. Nociceptors—Noxious Stimulus Detectors. Neuron. 2007;55:353–64.

8. Morgan EF, De Giacomo A, Gerstenfeld LC. Overview of skeletal repair (fracture healing and its assessment). Methods Mol Biol. 2014;1130:13–31.

9. Freeman KT, Koewler NJ, Jimenez-Andrade JM, Buus RJ, Herrera MB, Martin CD, et al. A fracture pain model in the rat: adaptation of a closed femur fracture model to study skeletal pain. Anesthesiology. 2008;108:473–83.

10. Gerstenfeld LC, Cullinane DM, Barnes GL, Graves DT, Einhorn TA. Fracture healing as a post-natal developmental process: molecular, spatial, and temporal aspects of its regulation. J Cell Biochem. 2003;88:873–84.

11. Chartier SR, Thompson ML, Longo G, Fealk MN, Majuta LA, Mantyh PW. Exuberant sprouting of sensory and sympathetic nerve fibers in nonhealed bone fractures and the generation and maintenance of chronic skeletal pain. Pain. 2014;155:2323–36.

12. Marinus J, Moseley GL, Birklein F, Baron R, Maihöfner C, Kingery WS, et al. Clinical features and pathophysiology of complex regional pain syndrome. Lancet Neurol. 2011;10:637–48.

13. Birklein F, Künzel W, Sieweke N. Despite clinical similarities there are significant differences between acute limb trauma and complex regional pain syndrome I (CRPS I). Pain. 2001;93:165–71.

14. Schindeler A, McDonald MM, Bokko P, Little DG. Bone remodeling during fracture repair: The cellular picture. Semin Cell Dev Biol. 2008;19:459–66.

15. Wang W, Jiang Q, Wu J, Tang W, Xu M. Upregulation of bone morphogenetic protein 2 (Bmp2) in dorsal root ganglion in a rat model of bone cancer pain. Mol Pain. 2019;15:1744806918824250.

16. Mitchell K, Shah JP, Dalgard CL, Tsytsikova LV, Tipton AC, Dmitriev AE, et al. Bone morphogenetic protein-2-mediated pain and inflammation in a rat model of posterolateral arthrodesis.

BMC Neurosci. 2016;17:80.

17. Nguyen V, Meyers CA, Yan N, Agarwal S, Levi B, James AW. BMP-2-induced bone formation and neural inflammation. J Orthop. 2017;14:252–6.
